# Supplementary material for: Evolutionary regimes determine the accuracy of epistasis inference from temporal genetic data
Source: bioRxiv. 2026 Jul 20:2026.07.16.739011. Preprint. [Version 1] doi: 10.64898/2026.07.16.739011 (PMC13419832; doi:10.64898/2026.07.16.739011)
Supplement: Supplementary file 1 [file NIHPP2026.07.16.739011v1-supplement-1.pdf]

bioRxiv preprint doi: https://doi.org/10.64898/2026.07.16.739011; this version posted July 20, 2026. The copyright holder for this preprint (which was not certified by peer review) is the author/funder, who has granted bioRxiv a license to display the preprint in perpetuity. It is made available under a CC-BY 4.0 International license.

Supplementary Information

bioRxiv preprint doi: https://doi.org/10.64898/2026.07.16.739011; this version posted July 20, 2026. The copyright holder for this preprint (which was not certified by peer review) is the author/funder, who has granted bioRxiv a license to display the preprint in perpetuity. It is made available under a CC-BY 4.0 International license.

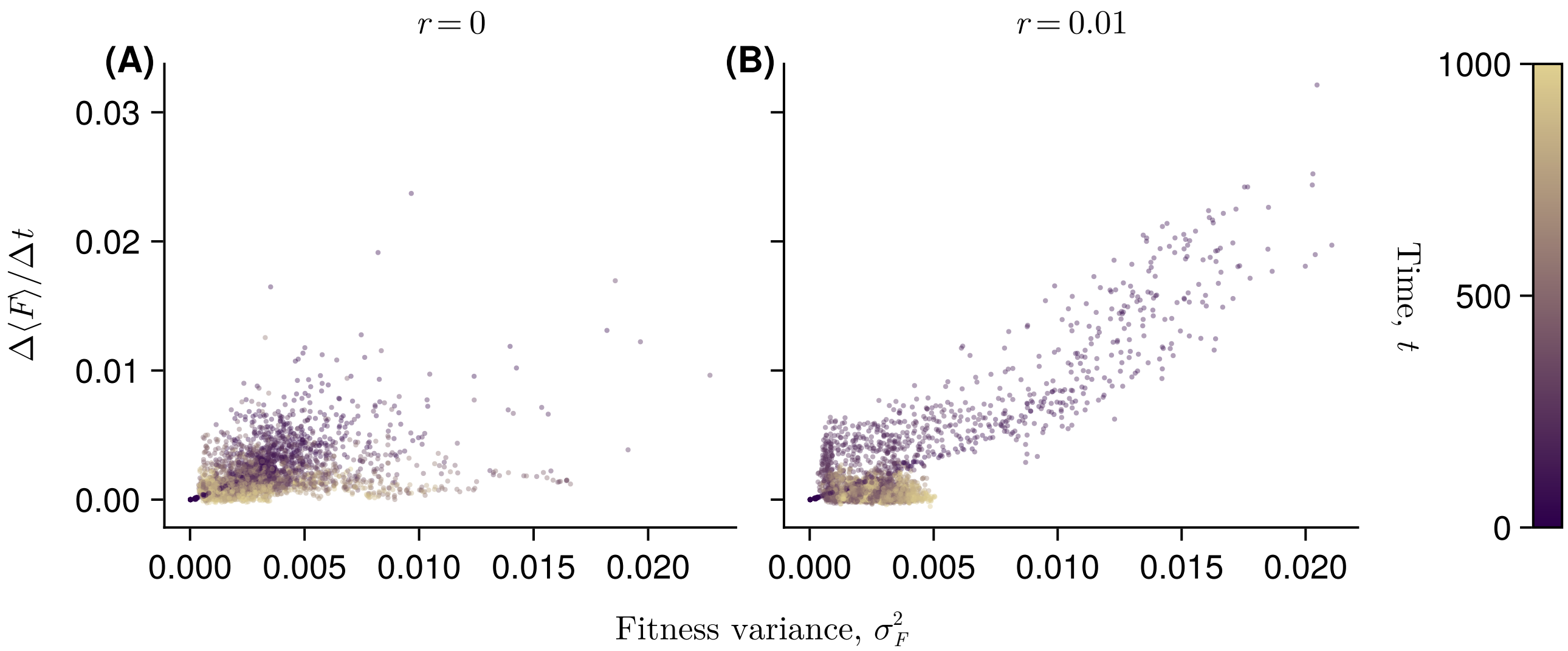

Supplementary Fig. S1. Shown are low-drift, strong-selection conditions ($\mu = 10^{-4}, N = 10^4$), where selection is the dominant driver of changes in mean fitness $\langle F\rangle$. The rate of mean fitness change, $\Delta\langle F\rangle/\Delta t$, is estimated from numerical changes over $\Delta t = 30$ generations. Each point represents the result from one of the 30 replicates. Panel **A** shows the asexual case ($r = 0$), whereas panel **B** shows frequent recombination ($r = 0.01$), which leads to a rapid increase in $\langle F\rangle$ and large σ_F^2. Overall, $\Delta\langle F\rangle/\Delta t$ and σ_F^2 are approximately linearly correlated, particularly at early generations.

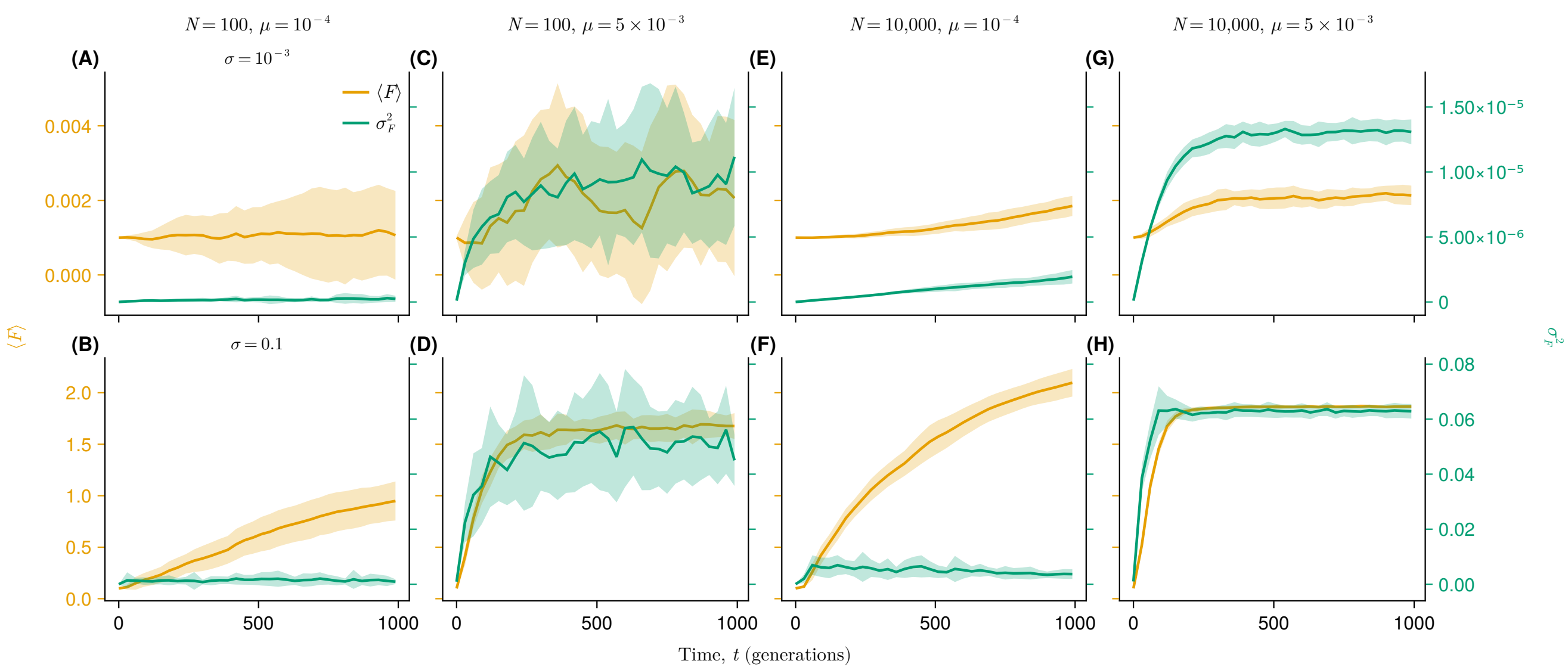

Supplementary Fig. S2. Same as above, these results are derived from asexual evolution ($r = 0$). Overall, smaller-σ cases (panels **A**, **C**, **E**, and **G**) maintain lower mean fitness and fitness variance, whereas larger-σ cases (panels **B**, **D**, **F**, and **H**) push the average fitness to higher values. This remains true even after normalizing the mean fitness by σ, i.e., using $\langle F\rangle/\sigma$, to remove the explicit σ-dependence of the fitness function. For example, the ratio of mean fitness between the large- and small-σ cases is approximately $1.5/0.002 = 750$, which is much larger than the ratio of the σ values themselves, $0.1/0.001 = 100$. This suggests that larger σ not only rescales fitness, but also changes the population dynamics by more efficiently amplifying high-fitness genotypes. The normalized fitness variance, $\mathrm{Var}(F)/\sigma^2 = \sigma_F^2/\sigma^2$, shows a more complex pattern. Strong selection can rapidly push genotypes toward fixation or extinction, thereby reducing standing diversity. Consequently, the normalized variance can be lower in the strong-selection case. For example, in a single-locus case, the fitness variance is proportional to $\sigma^2 x(1-x)$, which becomes small when the allele frequency x approaches either 0 or 1. This is consistent with the heterozygosity results: strong selection, especially at large N, can produce lower average heterozygosity H than weak selection because allele frequencies become more polarized.

bioRxiv preprint doi: https://doi.org/10.64898/2026.07.16.739011; this version posted July 20, 2026. The copyright holder for this preprint (which was not certified by peer review) is the author/funder, who has granted bioRxiv a license to display the preprint in perpetuity. It is made available under a CC-BY 4.0 International license.

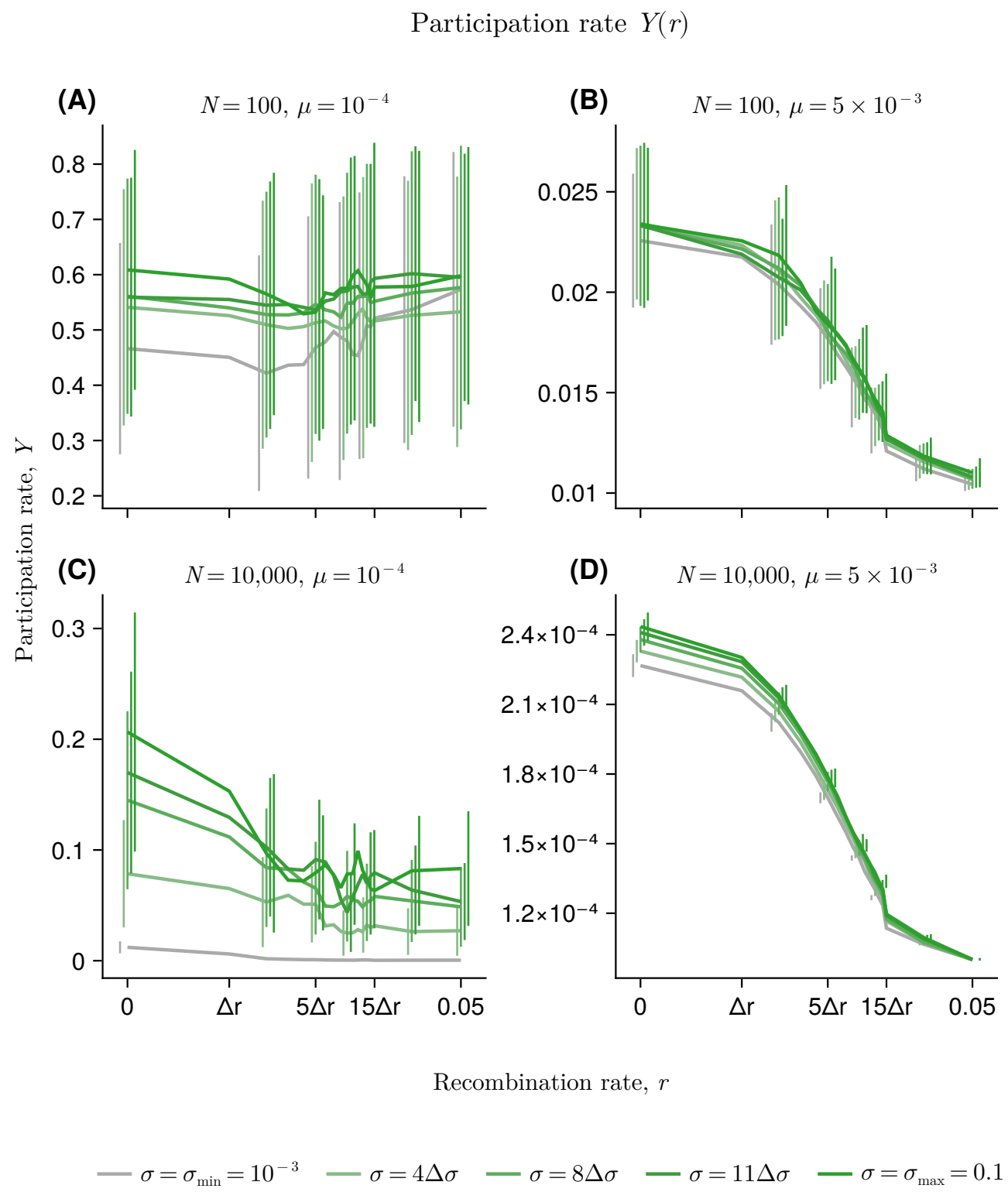

Supplementary Fig. S3. Effect of recombination rate on genotype concentration, quantified by the participation ratio $Y = \sum_\alpha x_\alpha^2$, where x_α is the frequency of genotype α. Each panel shows Y as a function of recombination rate r for a different combination of population size and mutation rate: $N = 100$, $\mu = 10^{-4}$ (**A**); $N = 100$, $\mu = 5\times10^{-3}$ (**B**); $N = 10^4$, $\mu = 10^{-4}$ (**C**); and $N = 10^4$, $\mu = 5\times10^{-3}$ (**D**). Lines and error bars show the mean and standard deviation across 30 replicate simulations. Darker green colors indicate larger values of σ, whereas gray colors indicate smaller values of σ. Overall, increasing recombination generally decreases Y, indicating reduced genotype condensation and an increased effective number of genotypes (see also the prior study that more quantitatively investigated the condensation of genotype using the metric Y (ref.[39]), which used a different setup: starting from diverse genotypes under absence of mutations).

bioRxiv preprint doi: https://doi.org/10.64898/2026.07.16.739011; this version posted July 20, 2026. The copyright holder for this preprint (which was not certified by peer review) is the author/funder, who has granted bioRxiv a license to display the preprint in perpetuity. It is made available under a CC-BY 4.0 International license.

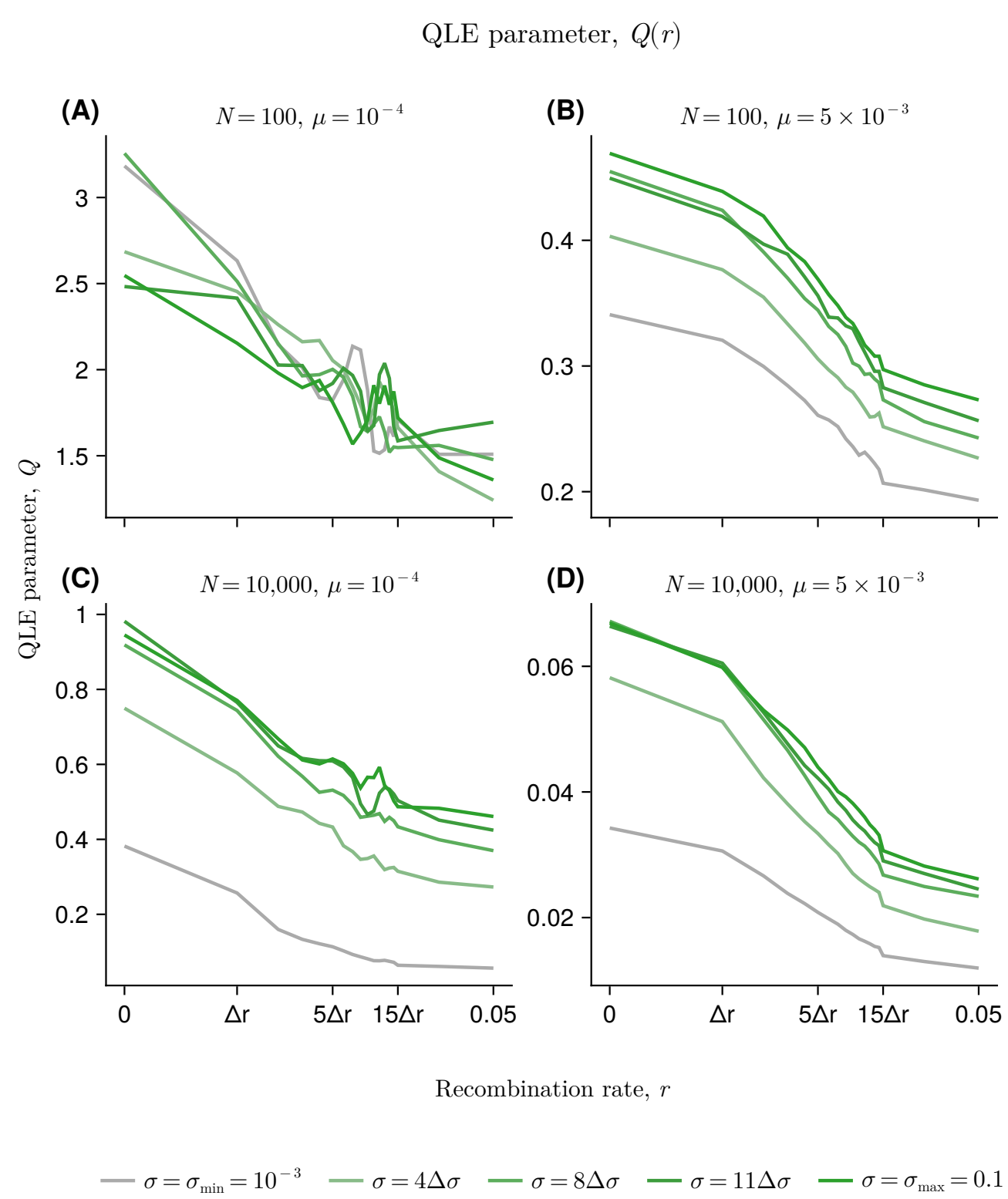

Supplementary Fig. S4. Effect of recombination rate on the QLE parameter Q, a metric that quantifies the average strength of pairwise associations among loci. Each panel shows Q as a function of recombination rate r for a different combination of N and μ (**A**-**D**). Lines and error bars show the mean and standard deviation across replicate simulations. Darker green colors indicate larger values of σ, whereas gray colors indicate smaller values of σ. As expected, frequent recombination generally reduces Q as recombination events break down the associations among loci. In contrast, increasing σ systematically increases Q, indicating that stronger selection or epistasis induces more nonrandom associations among loci.

bioRxiv preprint doi: https://doi.org/10.64898/2026.07.16.739011; this version posted July 20, 2026. The copyright holder for this preprint (which was not certified by peer review) is the author/funder, who has granted bioRxiv a license to display the preprint in perpetuity. It is made available under a CC-BY 4.0 International license.

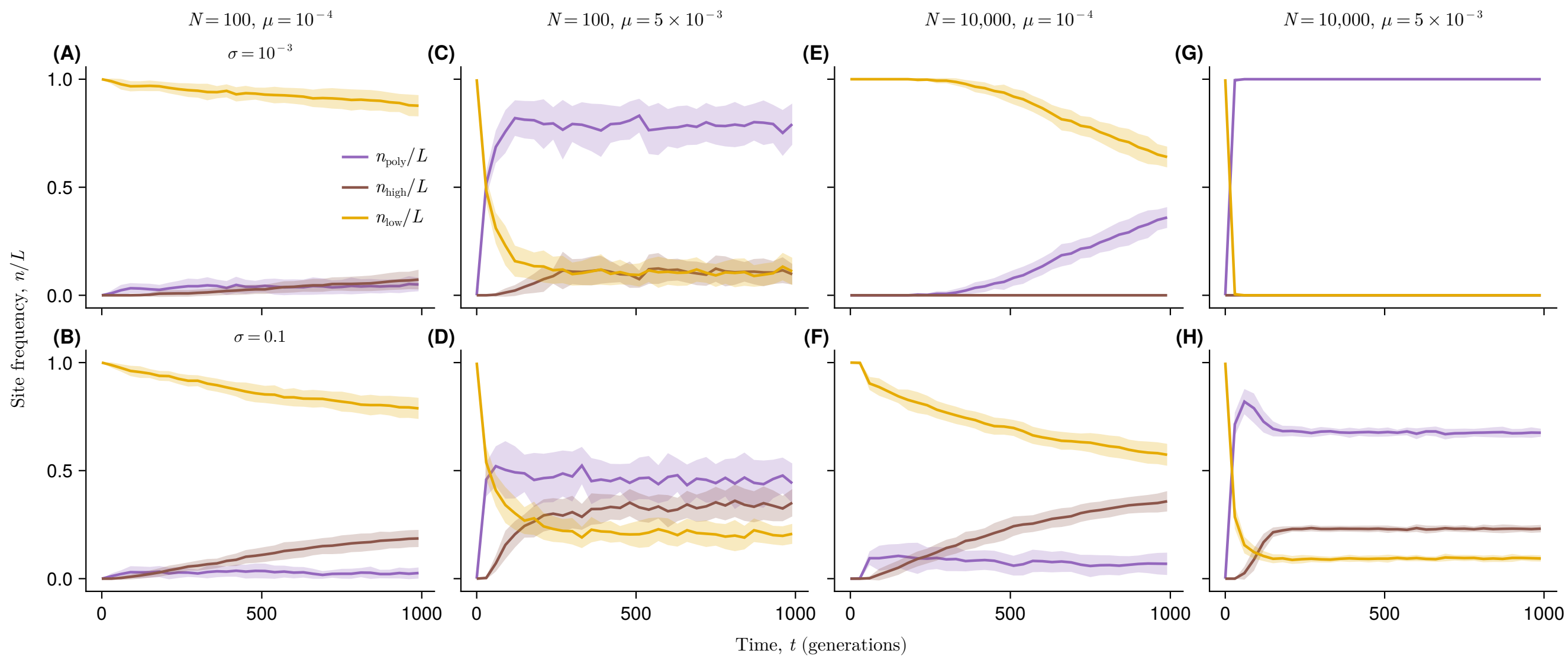

Supplementary Fig. S5. Here, n_{high} and n_{low} represent the number of sites at which the mutant allele frequency is above 90% or below 10%, respectively, and n_{poly} denotes the number of polymorphic sites with mutant frequency between 10% and 90%. Thus, n_{high}/L, n_{low}/L, and n_{poly}/L summarize the fractions of high-frequency, low-frequency, and polymorphic mutant sites, and sum to 1.0. Under weak selection (panels **A**, **C**, **E**, and **G**), mutant sites rarely reach high frequency, and larger mutation supply primarily increases n_{poly}. When μNL is small, polymorphism remains limited, whereas larger μNL generates more polymorphic sites. Under strong selection (panels **B**, **D**, **F**, and **H**), n_{high} increases and n_{low} declines, reflecting the rise of beneficial mutations or high-fitness genetic backgrounds. With low mutation supply, the dynamics are sweep-like, whereas with high mutation supply, many mutations segregate simultaneously and the system approaches a dynamic balance between recurrent mutation and selection.

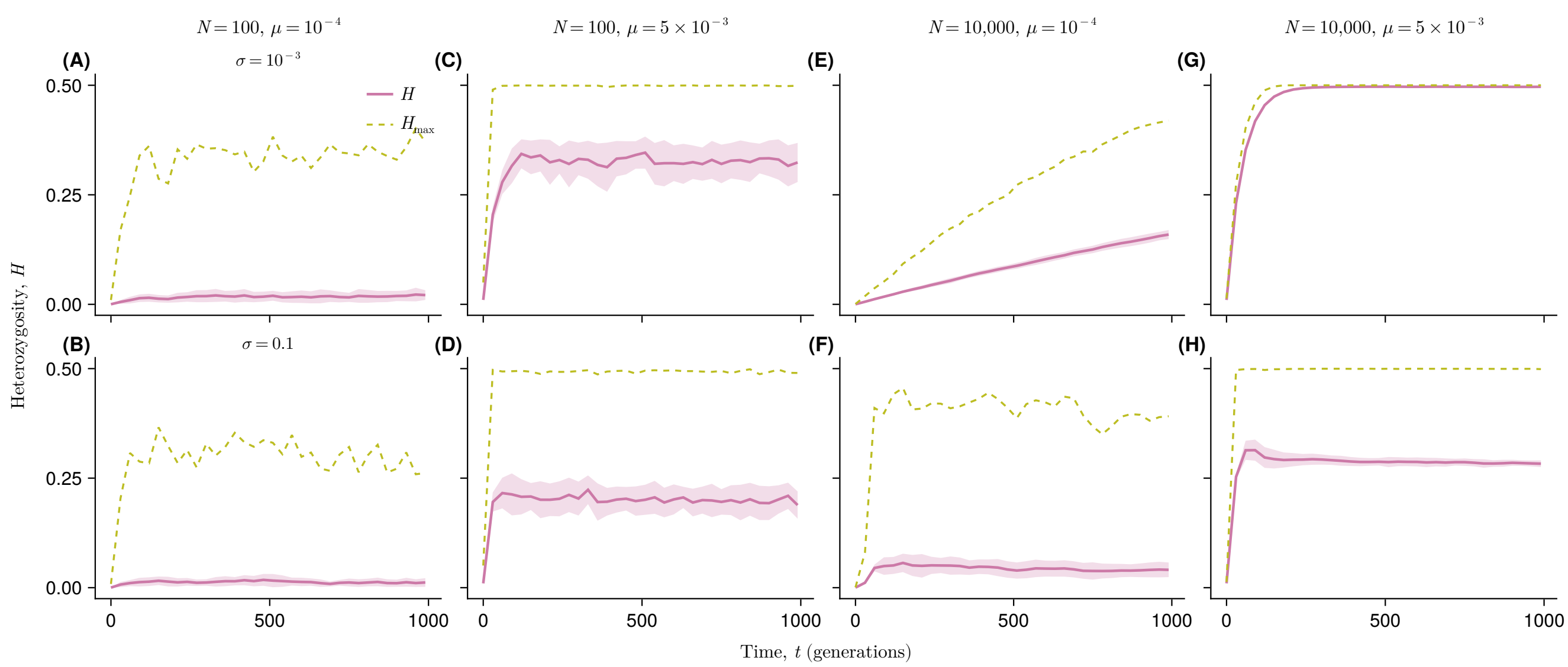

Supplementary Fig. S6. For small σ, evolution is close to neutral. The cases shown correspond to asexual evolution $(r = 0)$. As expected, the site averaged heterozygosity H remains low when both population size and mutation rate are small (panel **A**). With larger N and low μ, H increases slowly and reaches moderate values (panel **E**). At high μ, H rises rapidly (panels **C** and **G**), and when N is also large, H approaches its maximum value. Under strong selection, the dynamics depend strongly on mutation supply. When both N and μ are small, mutation supply is limited and H remains near zero (panel **B**). When μ is high, even in the small population, H rises rapidly but fluctuates around intermediate values, reflecting recurrent mutation together with drift and stochastic lineage dynamics (panel **D**). With large N aand low μ, mutation supply is larger but still much lower than in the high-μ case (panel **F**). In this regime, average H remains low even though individual sites can transiently reach high heterozygosity as sweeping mutations pass through intermediate frequency before approaching fixation. Finally, with both large N and high μ, recurrent mutation maintains substantial polymorphism, while strong selection drives some sites toward high or low frequency (panel **H**). Thus, some sites remain near intermediate frequency, whereas others become nearly fixed, consistent with competition among selected genetic backgrounds.

bioRxiv preprint doi: https://doi.org/10.64898/2026.07.16.739011; this version posted July 20, 2026. The copyright holder for this preprint (which was not certified by peer review) is the author/funder, who has granted bioRxiv a license to display the preprint in perpetuity. It is made available under a CC-BY 4.0 International license.

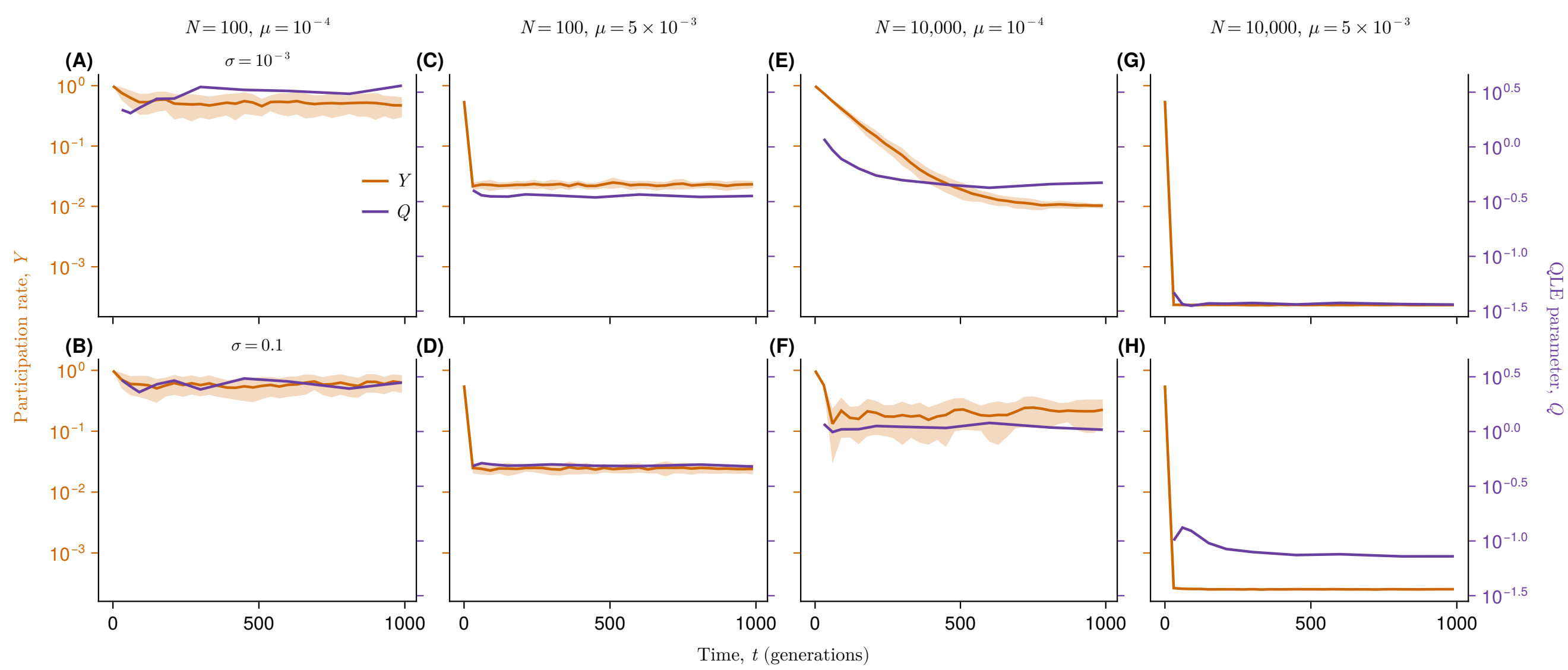

Supplementary Fig. S7. The cases shown correspond to $r = 0$ case. $Y = \sum_\alpha x_\alpha^2$, where x_α is the frequency of genotype α, serves as a genotype-concentration parameter. Its inverse, $1/Y$, represents the effective number of genotypes. We also define $Q = \sum_{i<j}\langle\chi_{ij}\rangle / \sum_{i<j}\langle\chi_{ii}\chi_{jj}\rangle$, where χ_{ij} is the second-order cumulant and $\langle\cdot\rangle$ denotes the average over independent simulations with the same underlying fitness parameters. Thus, Q measures LD relative to single-locus diversity. The difference is that Y measures concentration at the genotype level, whereas Q measures correlated allele structure across loci. These quantities are expected to behave similarly when genotype-level concentration creates linkage disequilibrium among many loci. Under neutral evolution, $Y \sim 1/(1 + 2\mu NL)$, and this approximation matches the steady-state values of Y for the weak-selection cases with $\sigma = 10^{-3}$ (panels **A**, **C**, **E**, and **G**). Selection can increase Y by amplifying high-fitness genotypes; however, in our simulations, the effect of selection on Y was more subtle (panels **B**, **D**, **F**, and **H**). As expected, Q behaves similarly to Y. However, Q is generally higher in the larger-σ cases, even when Y is close to $1/N$ (for example, compare panels **G** and **H**). This suggests elevated nonrandom association between loci under stronger selection. Overall, Y and Q show qualitatively similar behavior, suggesting elevated LD is often associated with genotype concentration. However, elevated Q when Y is small suggests also that nonrandom associations can also persist even when the population is diversifed ($Y \simeq 1/N$), likely due to selection and epistasis.

bioRxiv preprint doi: https://doi.org/10.64898/2026.07.16.739011; this version posted July 20, 2026. The copyright holder for this preprint (which was not certified by peer review) is the author/funder, who has granted bioRxiv a license to display the preprint in perpetuity. It is made available under a CC-BY 4.0 International license.

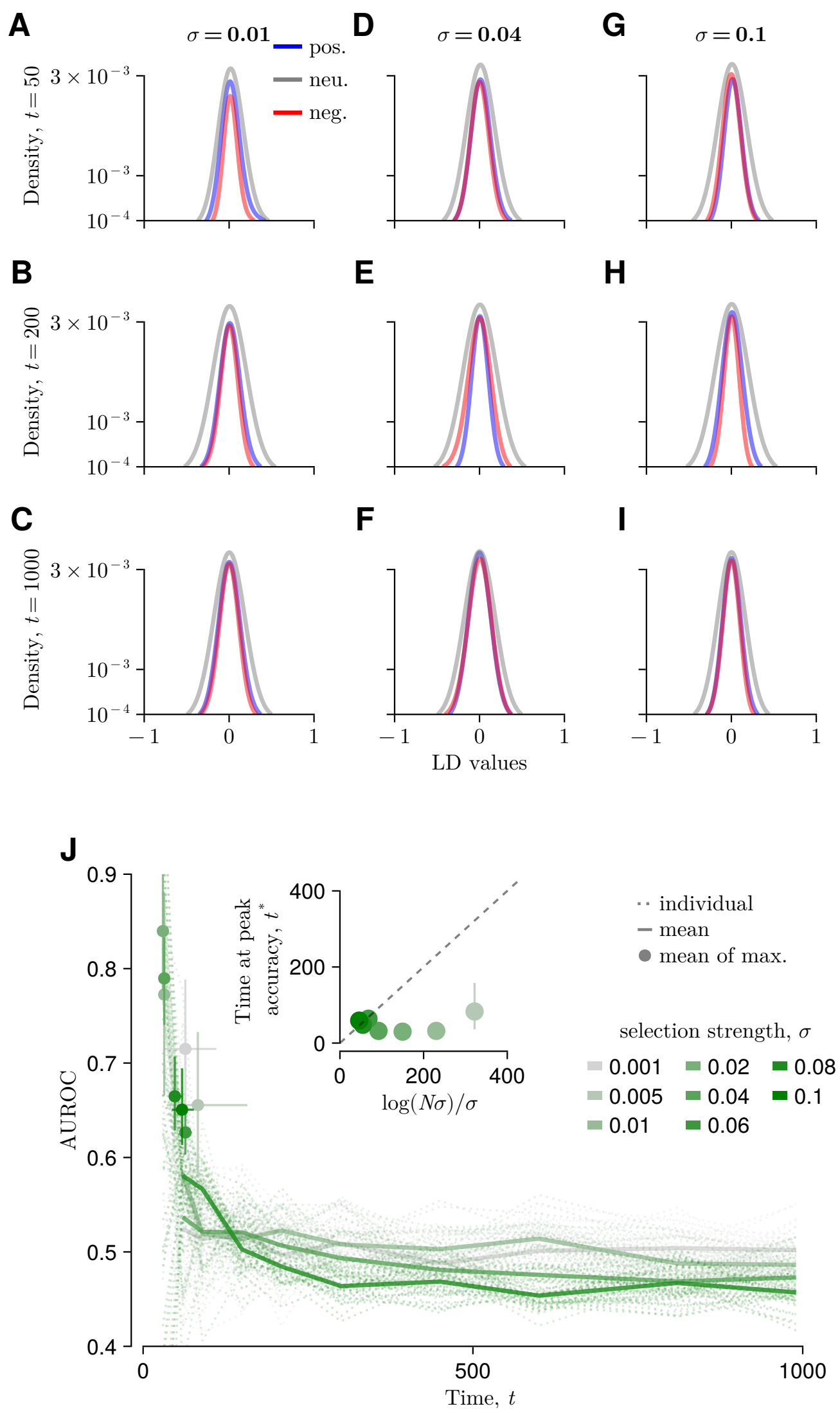

Supplementary Fig. S8. Distribution of LD for frequent recombination ($r = 0.01$) case. A–I Same analysis as **Fig. 3A-I**, but for the $N = 10^3$ and $r = 0.01$ case rather than $r = 0$. Across all time points and σ values, the distributions collapse onto a normal distribution with zero mean. The AUROC value, used as accuracy metric, remains low across all times and σ values; therefore, unlike the $r = 0$ case, the peak time t^* is not simply related to $\log(N\sigma)/\sigma$ (**J**).

bioRxiv preprint doi: https://doi.org/10.64898/2026.07.16.739011; this version posted July 20, 2026. The copyright holder for this preprint (which was not certified by peer review) is the author/funder, who has granted bioRxiv a license to display the preprint in perpetuity. It is made available under a CC-BY 4.0 International license.

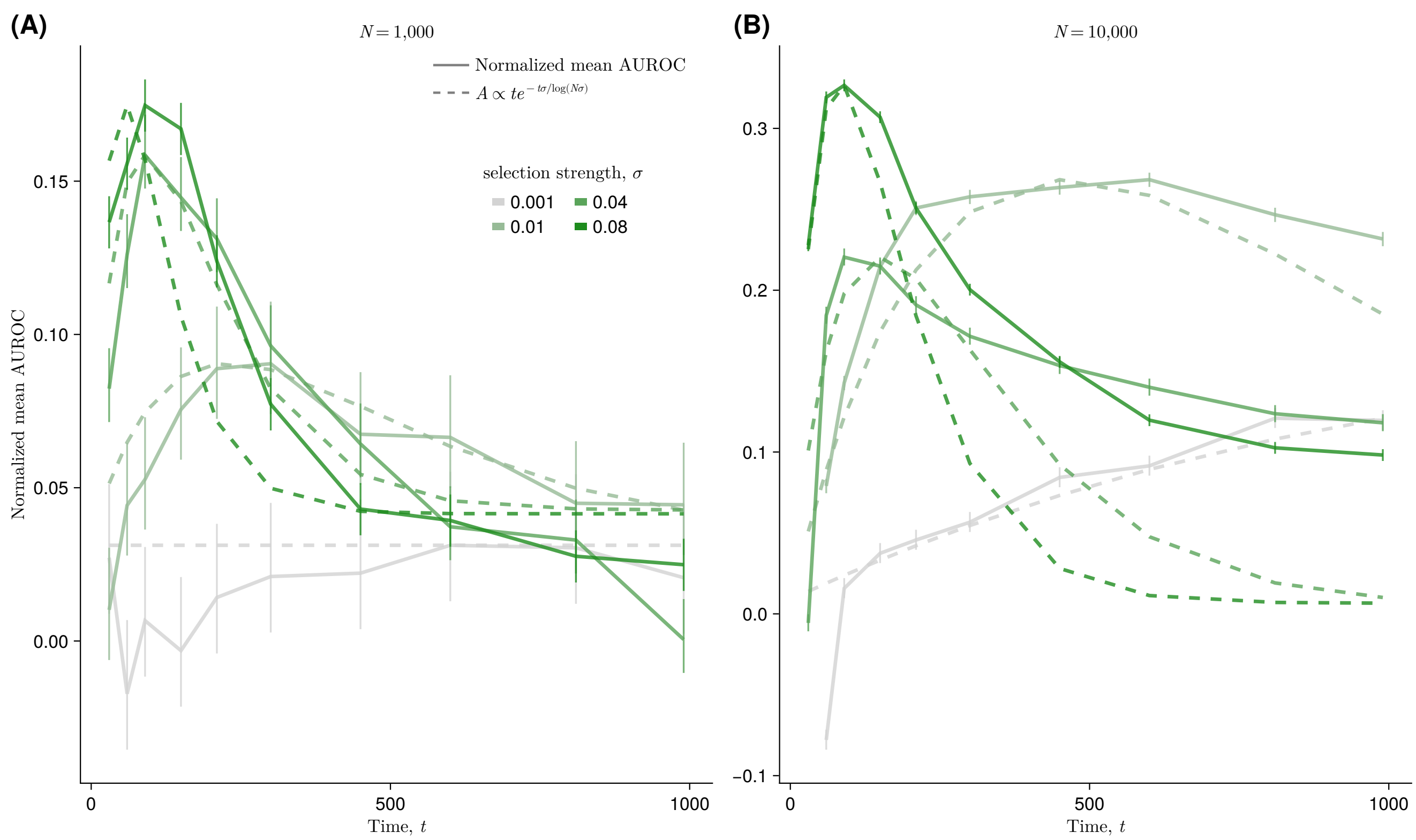

Supplementary Fig. S9. Fitting of the mean AUROC trajectories using the phenomenological expression of accuracy Eq. (10) The presented mean AUROC trajectories are normalized by subtracting 0.5 from the raw AUROC values. The raw AUROC values are identical to those shown in **(A) Fig. 3J** and **(B) Fig. S11J** for the cases $N = 10^3$ and $N = 10^4$, respectively. The amplitude of the phenomenological accuracy function $A(t)$ is estimated such that the peak values of $A(t)$ and the observed mean values match. The overall profile of the accuracy trajectory, as well as the time at which the accuracy reaches its maximum, is well characterized by the phenomenological function. The error bars represent the 95% confidence intervals estimated across 100 inference results.

bioRxiv preprint doi: https://doi.org/10.64898/2026.07.16.739011; this version posted July 20, 2026. The copyright holder for this preprint (which was not certified by peer review) is the author/funder, who has granted bioRxiv a license to display the preprint in perpetuity. It is made available under a CC-BY 4.0 International license.

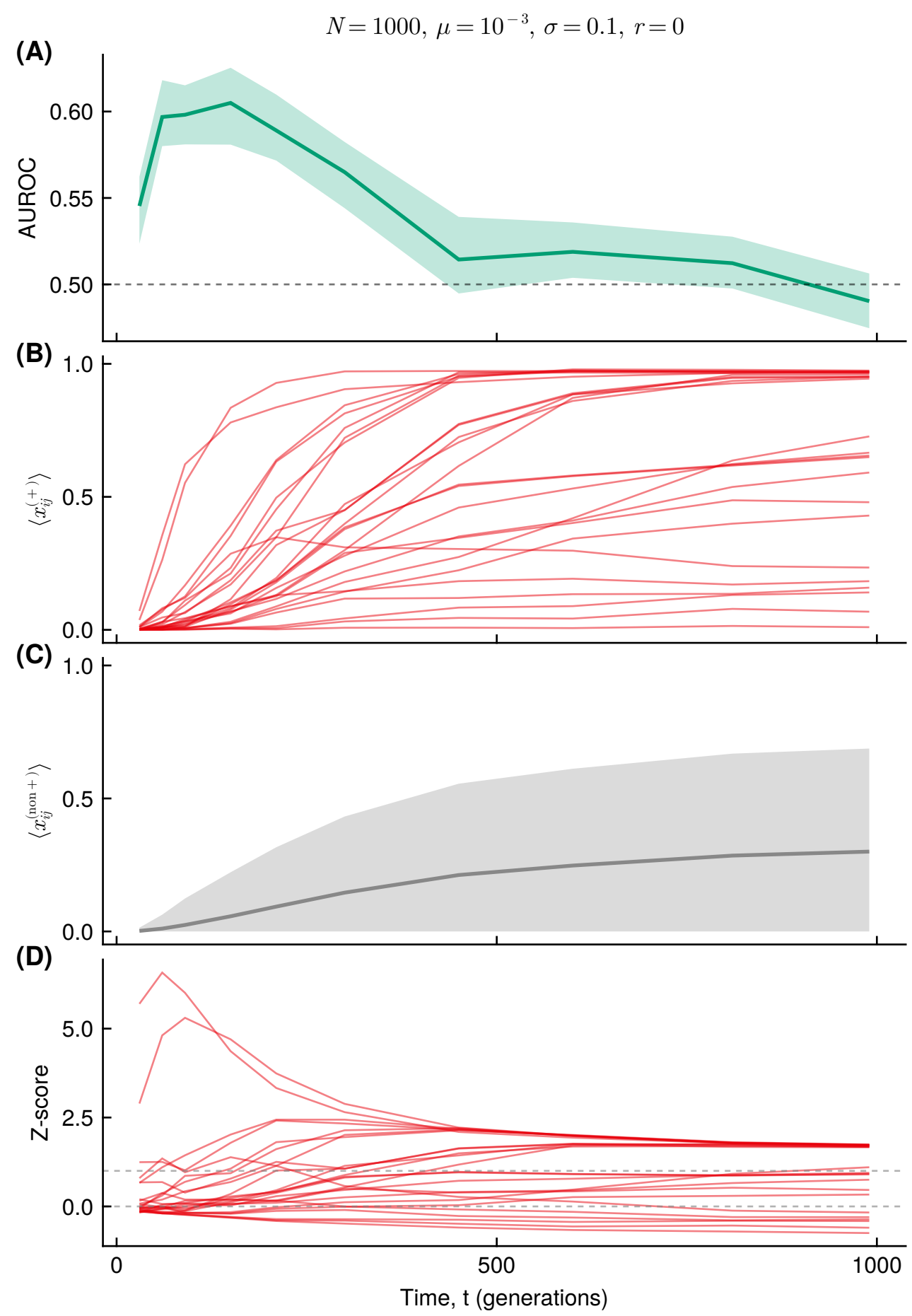

Supplementary Fig. S10. Dynamics of pairwise frequencies underlying the transient accuracy of LD-based epistasis detection. Results are shown for $N = 10^3$, $\mu = 10^{-3}$, $r = 0$, and $\sigma = 0.1$. **(A)** AUROC for detecting positive epistatic interactions using the LD-based method, reproduced from **Fig. 4**. **(B)** Mean pairwise frequency of mutation pairs with positive epistasis, denoted $\langle x_{ij}^{(+)} \rangle$. **(C)** Mean pairwise frequency of pairs without positive epistasis, denoted $\langle x_{ij}^{(\mathrm{non+})} \rangle$. Solid lines and shaded regions indicate the mean and standard deviation across mutation pairs and 30 replicate simulations. **(D)** Standardized separation between positively epistatic pairs and the background, $Z = \frac{\langle x_{ij}^{(+)} \rangle - \langle x_{ij}^{(\mathrm{non+})} \rangle}{\sqrt{\mathrm{Var}(x_{ij}^{(\mathrm{non+})})}}$, which quantifies how strongly positively epistatic pairs are enriched relative to non-positive pairs. The early increase in this separation explains the initial improvement in LD-based detection accuracy, whereas its later decrease reflects the accumulation of background linkage among non-positive pairs.

bioRxiv preprint doi: https://doi.org/10.64898/2026.07.16.739011; this version posted July 20, 2026. The copyright holder for this preprint (which was not certified by peer review) is the author/funder, who has granted bioRxiv a license to display the preprint in perpetuity. It is made available under a CC-BY 4.0 International license.

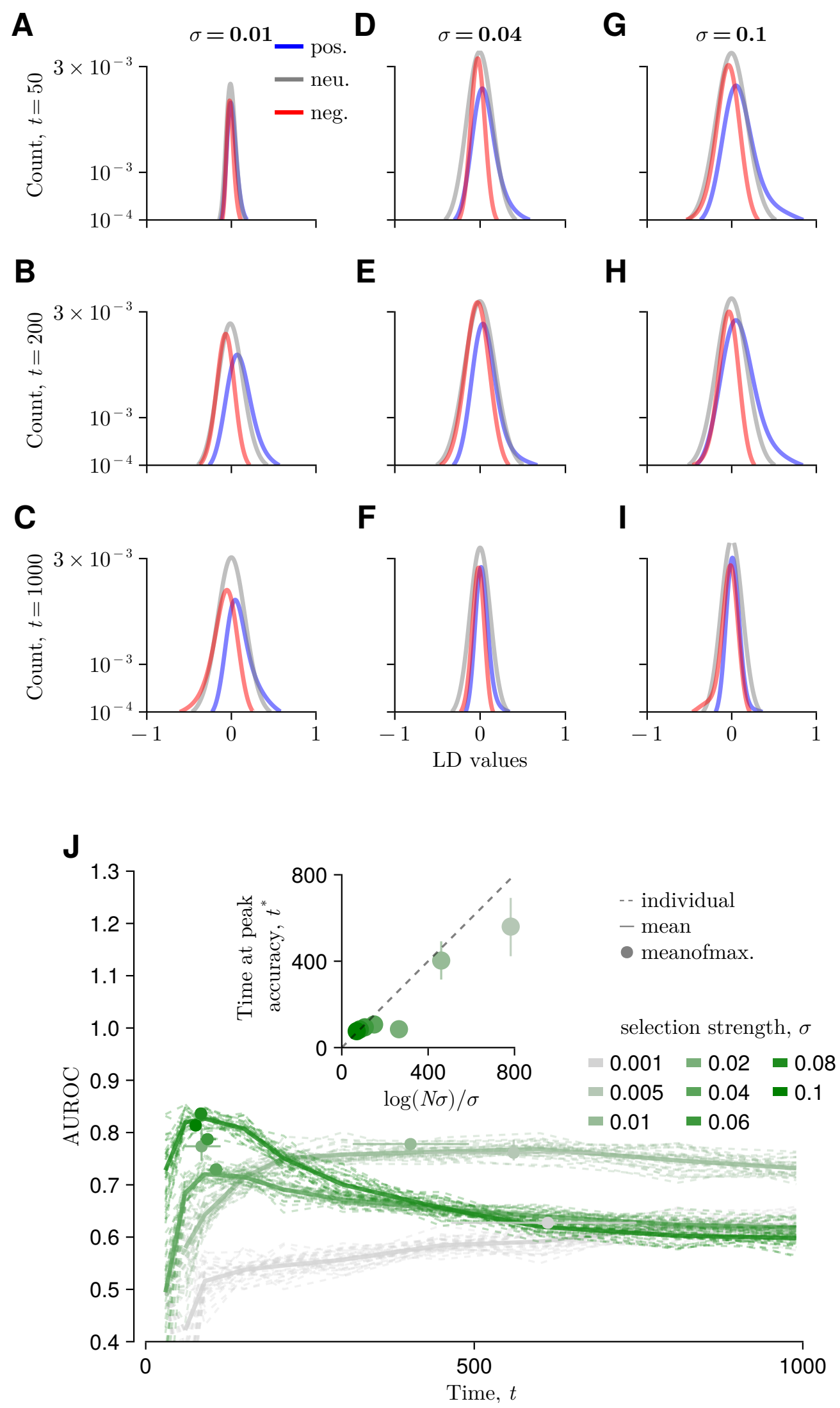

Supplementary Fig. S11. Distribution of LD for larger N case. A–I Distribution of LD values over time and selection strength σ for the $N = 10^4$ and $r = 0$ case. The qualitative dependence on time and σ is the same as in the $N = 10^3$ simulations: distributions become more separated at intermediate times and progressively overlap as time advances. The time window in which this separation appears also depends on σ. The average AUROC values based on LD show the same trend as in the $N = 10^3$ case: as σ increases, the AUROC peak occurs earlier, and the peak time is approximately proportional to $\log(N\sigma)/\sigma$ (**J**).

bioRxiv preprint doi: https://doi.org/10.64898/2026.07.16.739011; this version posted July 20, 2026. The copyright holder for this preprint (which was not certified by peer review) is the author/funder, who has granted bioRxiv a license to display the preprint in perpetuity. It is made available under a CC-BY 4.0 International license.

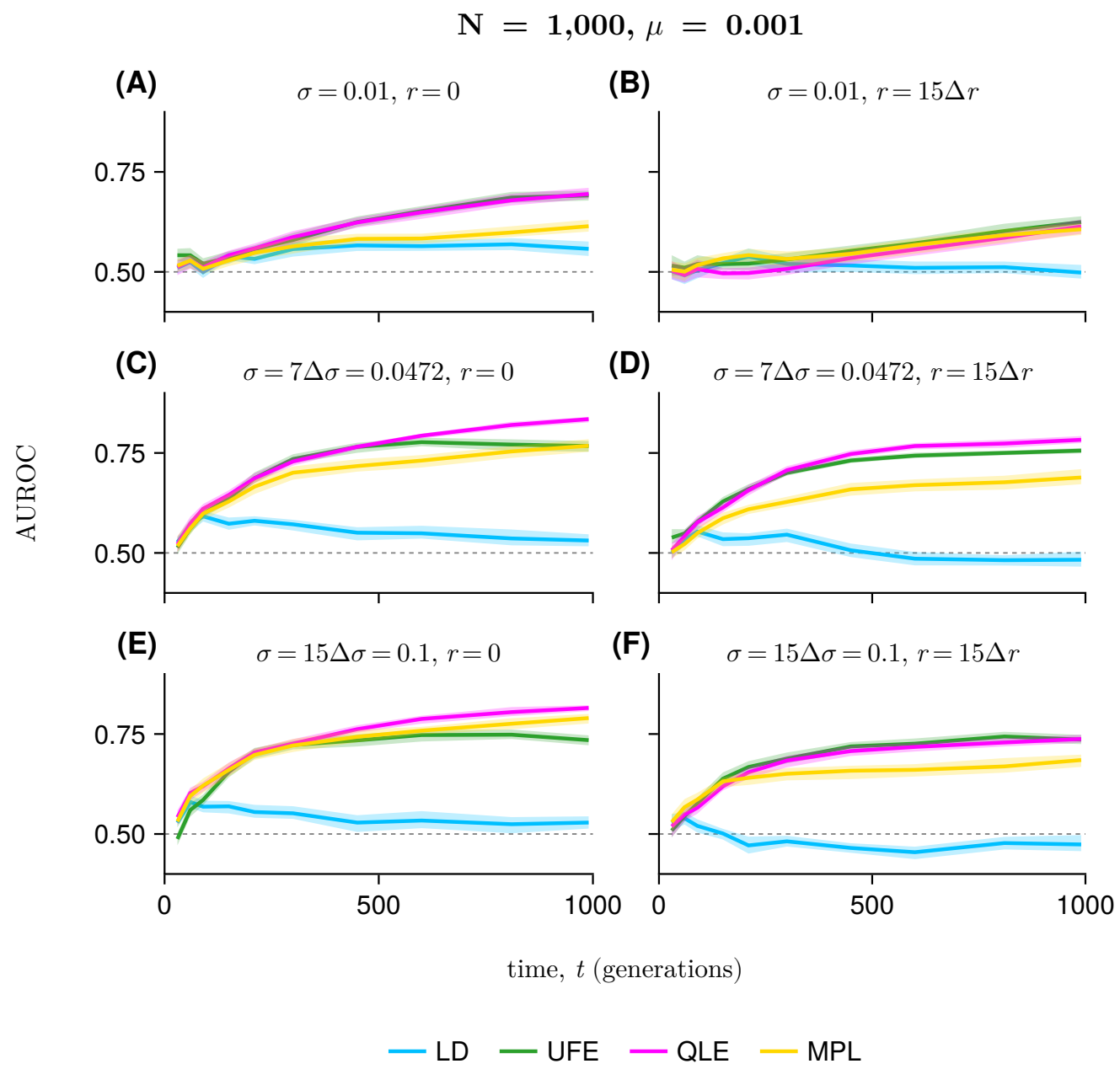

Supplementary Fig. S12. Detection accuracy for negative epistatic interactions as a function of time. This figure corresponds to **Fig. 4** , which is for the positive epistasis case. Population size and mutation rate are fixed at $N = 1000$ and $\mu = 10^{-3}$. The left column shows simulations without recombination, $r = 0$, and the right column shows simulations with high recombination frequency, $r = 0.01$. Overall, the qualitative patterns are similar to those observed for positive epistasis: accuracy is lower under weak selection, increases with selection strength, and is generally reduced by recombination. AUROC values are slightly lower for negative epistasis than for positive epistasis cases.

bioRxiv preprint doi: https://doi.org/10.64898/2026.07.16.739011; this version posted July 20, 2026. The copyright holder for this preprint (which was not certified by peer review) is the author/funder, who has granted bioRxiv a license to display the preprint in perpetuity. It is made available under a CC-BY 4.0 International license.

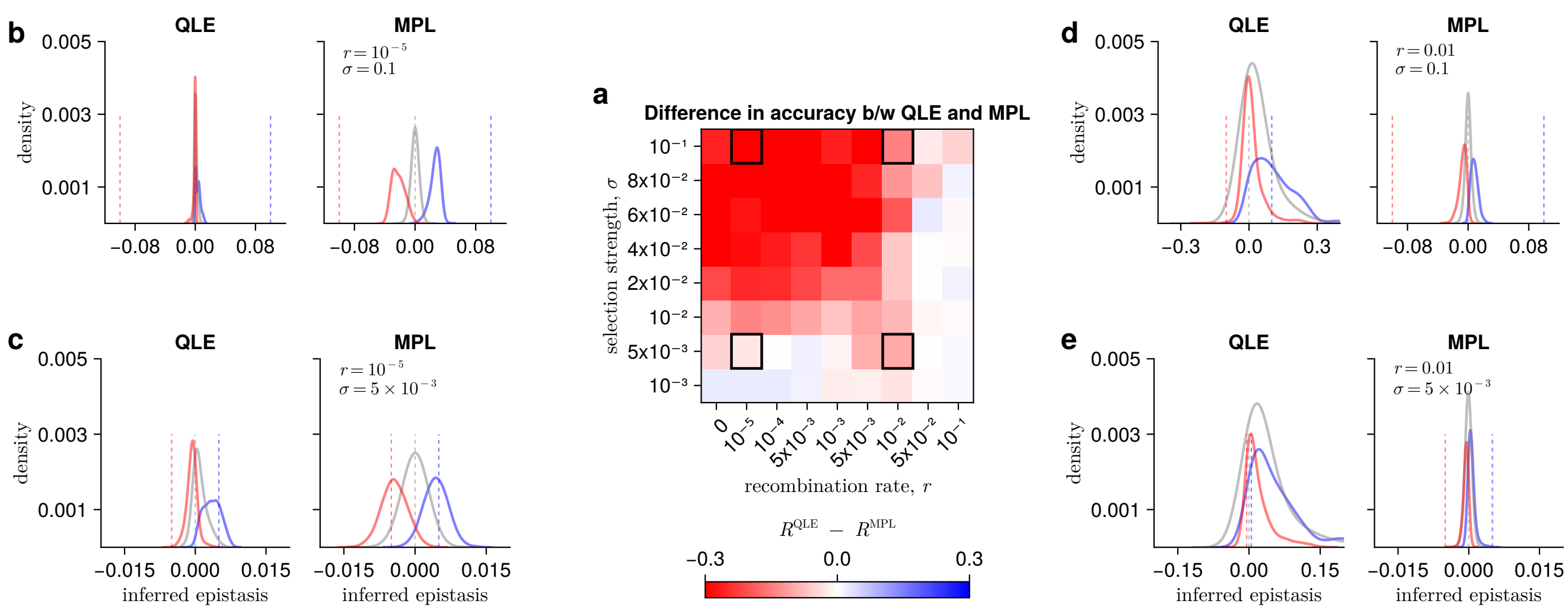

Supplementary Fig. S13. Results of inferred epistasis from WF simulations with $N = 10^4$ and $\mu = 10^{-3}$ using the MPL and QLE methods, visualized through the difference in Pearson's R between MPL and QLE ($R^{\mathrm{QLE}} - R^{\mathrm{MPL}}$). In this larger-$N$ setting, MPL outperforms QLE across most parameter regimes. The distributions for positive, negative, and neutral epistasis are more clearly separated, especially in the low-recombination regime, when using MPL.

bioRxiv preprint doi: https://doi.org/10.64898/2026.07.16.739011; this version posted July 20, 2026. The copyright holder for this preprint (which was not certified by peer review) is the author/funder, who has granted bioRxiv a license to display the preprint in perpetuity. It is made available under a CC-BY 4.0 International license.

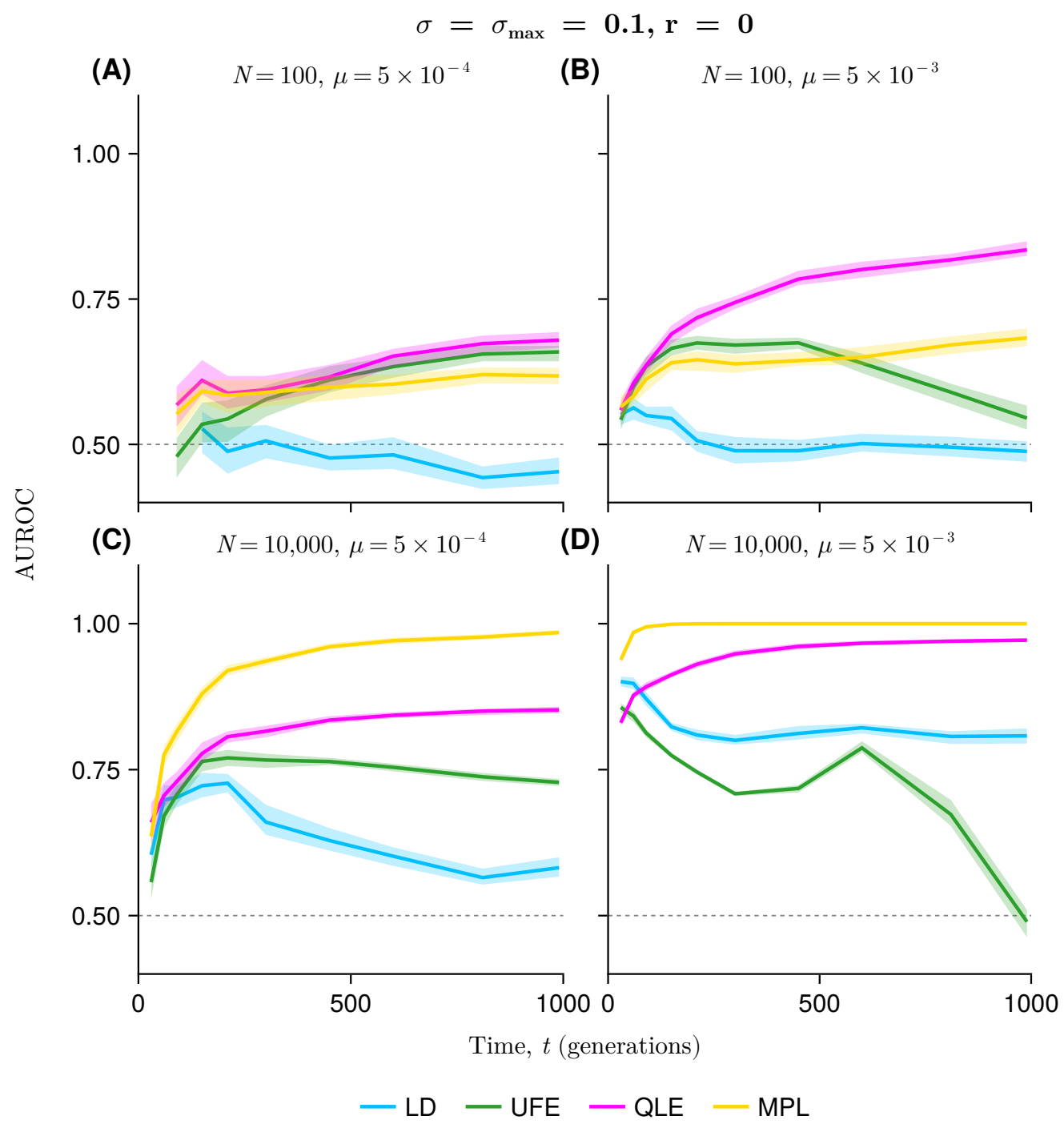

Supplementary Fig. S14. Time dependence of epistasis inference accuracy for representative combinations of population size and mutation rate under strong selection without recombination. Here, $\sigma = 0.1$ and $r = 0$. Each panel shows AUROC over time for the LD, UFE, QLE, and MPL methods. Panels correspond to $N = 100$, $\mu = 5 \times 10^{-4}$ (**A**); $N = 100$, $\mu = 5 \times 10^{-3}$ (**B**); $N = 10^4$, $\mu = 5 \times 10^{-4}$ (**C**); and $N = 10^4$, $\mu = 5 \times 10^{-3}$ (**D**). The relative performance of QLE and MPL depends on population size N. In smaller populations, QLE tends to reach higher accuracy than MPL, whereas in larger N, MPL generally outperforms QLE. At high μ and large N values, QLE and MPL become more similar in performance. UFE shows a distinct behavior; its accuracy can decrease over time, especially at high μ.

bioRxiv preprint doi: https://doi.org/10.64898/2026.07.16.739011; this version posted July 20, 2026. The copyright holder for this preprint (which was not certified by peer review) is the author/funder, who has granted bioRxiv a license to display the preprint in perpetuity. It is made available under a CC-BY 4.0 International license.

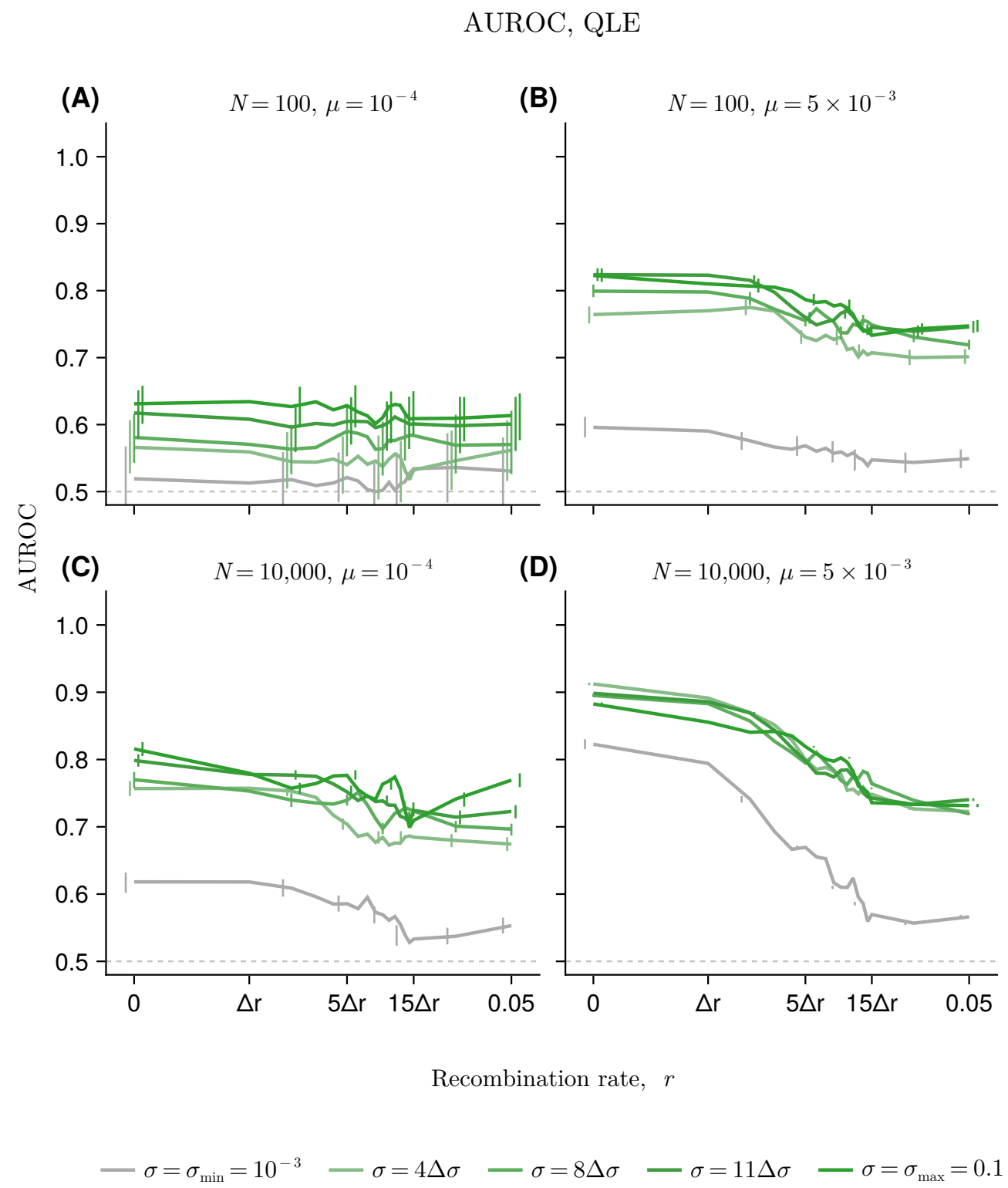

Supplementary Fig. S15. Effect of recombination rate on epistasis-inference accuracy for the QLE method. Each panel shows AUROC as a function of recombination rate r for $N = 100$, $\mu = 10^{-4}$ (**A**); $N = 100$, $\mu = 5 \times 10^{-3}$ (**B**); $N = 10^4$, $\mu = 10^{-4}$ (**C**); and $N = 10^4$, $\mu = 5 \times 10^{-3}$ (**D**). Lines and error bars show the mean and standard deviation across 30 replicate simulations. Across parameter regimes, QLE accuracy generally decreases as recombination rate increases, consistent with the reduction of associations among loci. An increase in the recombination decreases the accuracy and eventually the AUROC values from $N = 100$ and $N = 10^4$ become comparable at the highest r value, even though the AUROC value is significantly higher for the $N = 10^4$ case than the $N = 100$ case at $r = 0$. We also confirmed that the recombination does not impact the accuracy of selection inference.

bioRxiv preprint doi: https://doi.org/10.64898/2026.07.16.739011; this version posted July 20, 2026. The copyright holder for this preprint (which was not certified by peer review) is the author/funder, who has granted bioRxiv a license to display the preprint in perpetuity. It is made available under a CC-BY 4.0 International license.

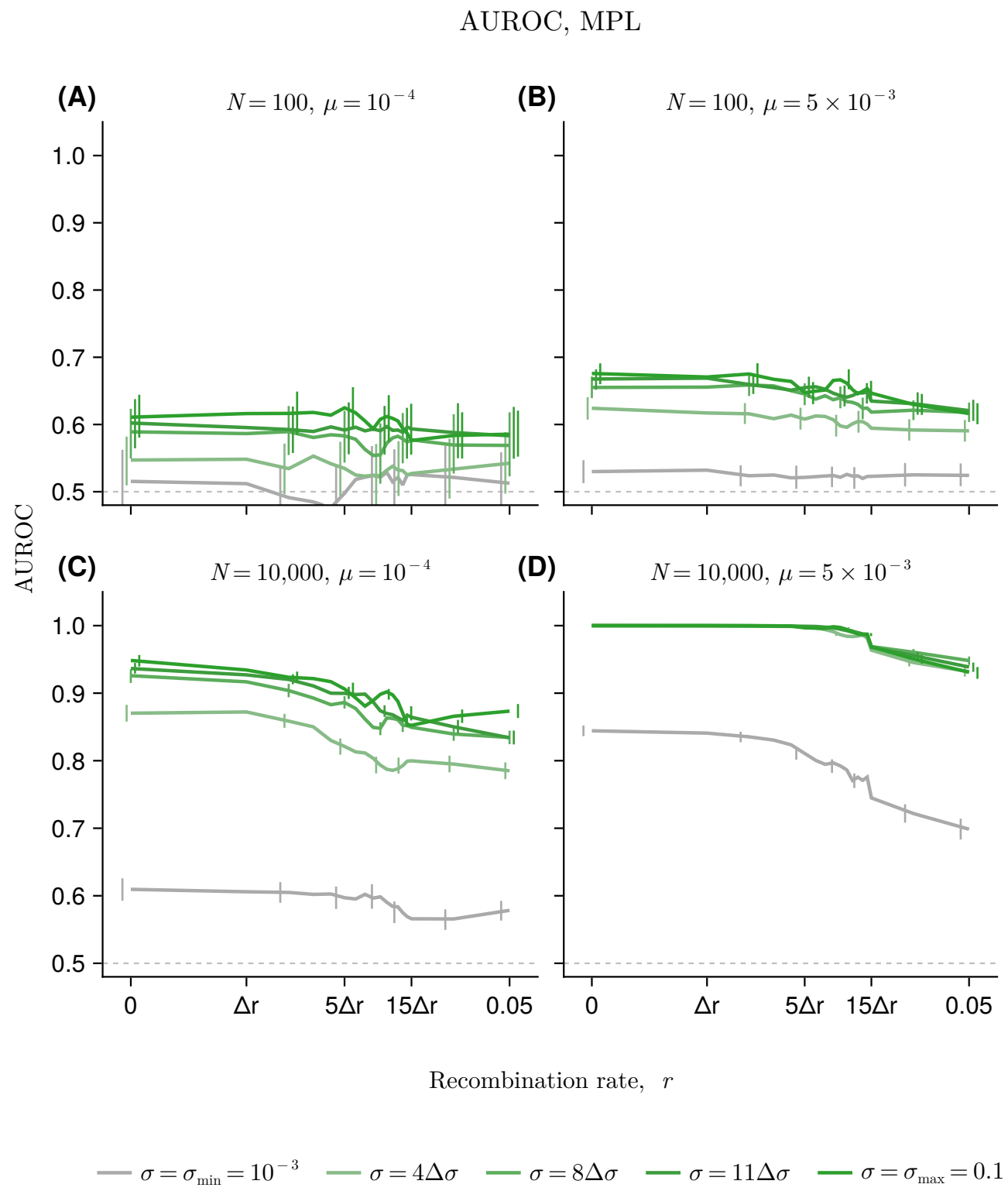

Supplementary Fig. S16. Effect of recombination rate on epistasis-inference accuracy for the MPL method. MPL accuracy typically depends on the σ value; a higher σ value results in a higher AUROC value. Recombination can also reduce accuracy for the MPL case.

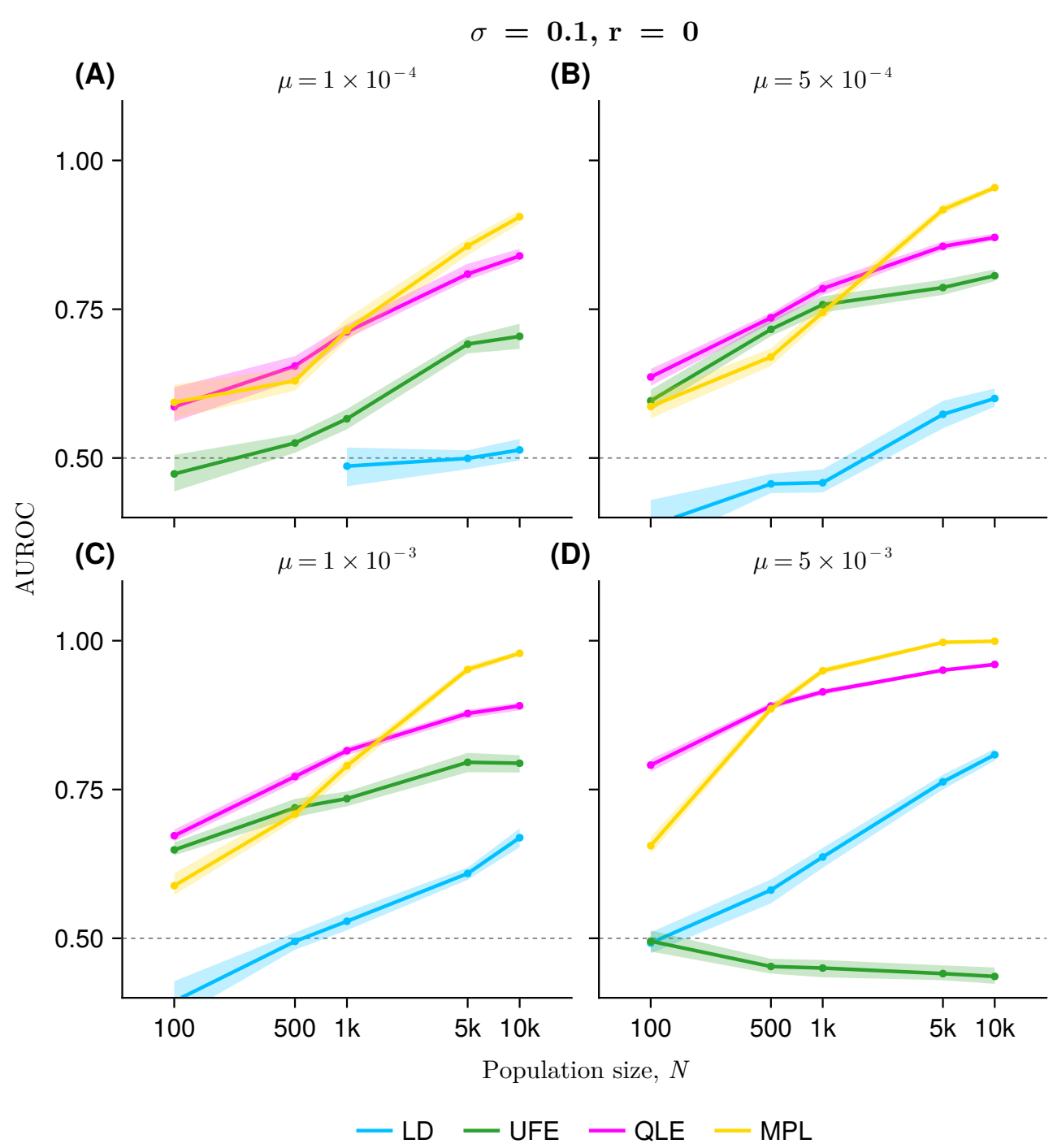

Supplementary Fig. S17. Effect of population size and mutation rate on detection accuracy for negative epistatic interactions. This figure corresponds to **Fig. 7**, which is the case for positive epistasis. Here, $\sigma = 0.1$ and $r = 0$. The population-size dependence is also qualitatively similar to the positive-epistasis case. Accuracy generally increases with N. The relative performance of the methods is also largely conserved, with QLE performing relatively well in smaller populations and MPL improving more strongly in larger populations. Absolute AUROC values are slightly lower than for positive epistasis overall.

bioRxiv preprint doi: https://doi.org/10.64898/2026.07.16.739011; this version posted July 20, 2026. The copyright holder for this preprint (which was not certified by peer review) is the author/funder, who has granted bioRxiv a license to display the preprint in perpetuity. It is made available under a CC-BY 4.0 International license.

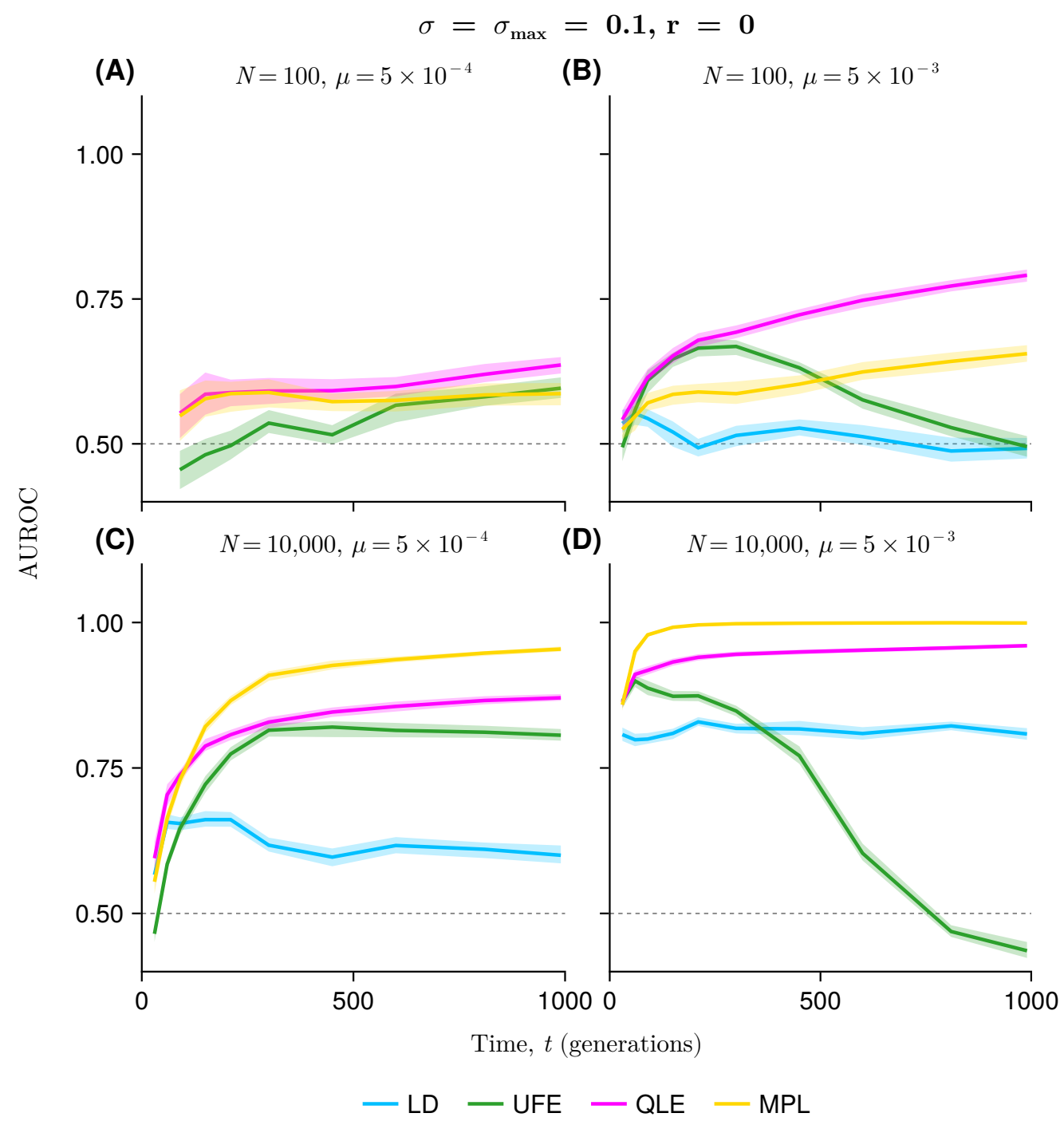

Supplementary Fig. S18. Time dependence of the detection accuracy for negative epistasis under representative N and μ combinations. This result corresponds to **Fig. S14**, which is for positive epistasis. The LD result is not shown for the $N = 100$, $\mu = 5 \times 10^{-4}$ case because no detectable mutation pairs passed the pair-frequency threshold used for the LD-based analysis. Overall, the time-dependent patterns and relative performance of the methods are similar to those observed for positive epistasis, although the absolute AUROC values are slightly lower. A notable difference is that the early-time peak characteristic of LD-based detection for positive epistasis becomes unclear for negative epistasis.

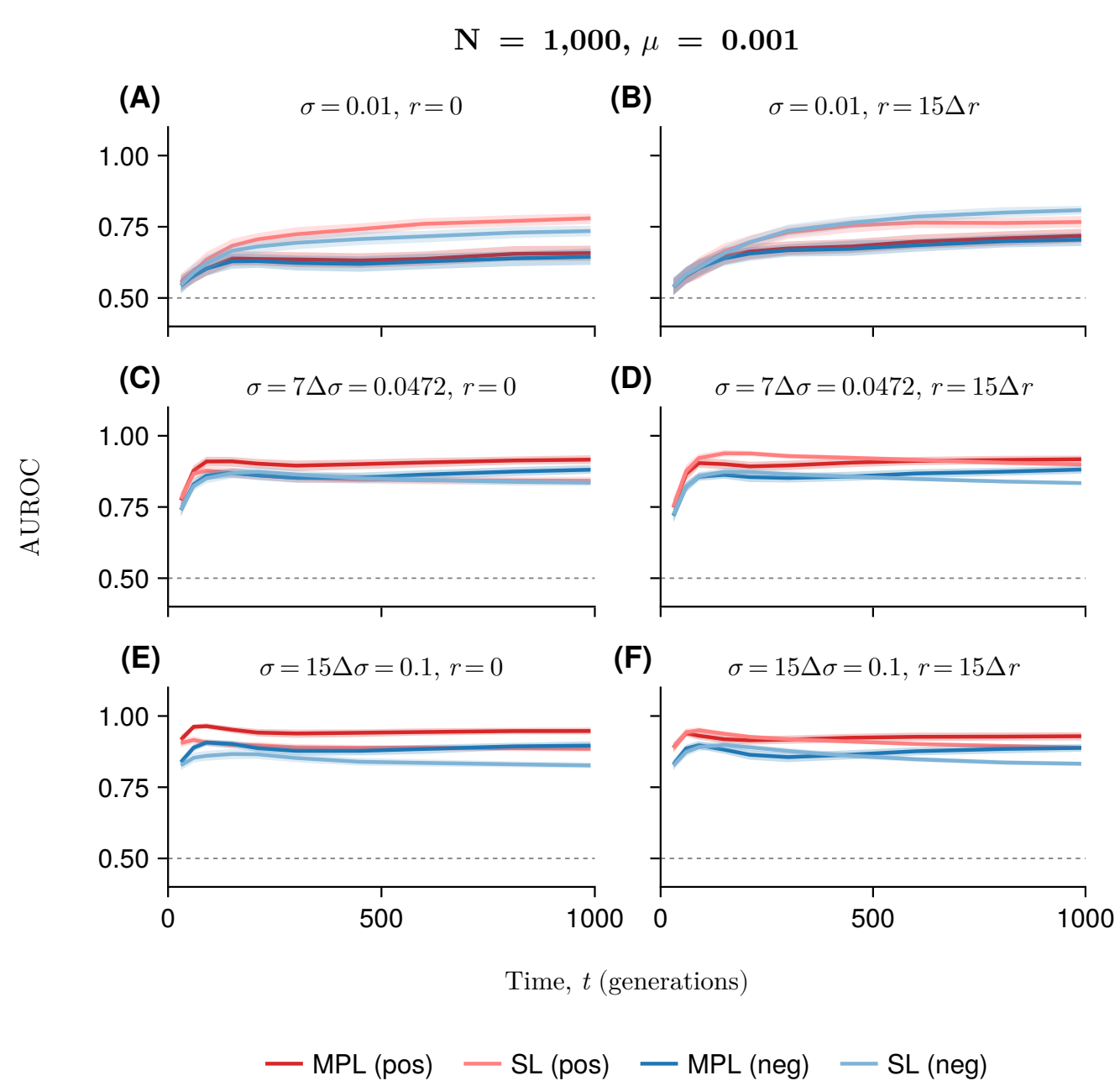

Supplementary Fig. S19. Selection-inference accuracy over time. Population size and mutation rate are fixed at $N = 1000$ and $\mu = 10^{-3}$. Here we focused on asexual (left) and frequent recombination (right) cases. The mean (bold line) and standard deviation (shaded ribbon) values are estimated from 30 replicates. Selection inference becomes more accurate as selection strength increases, consistent with the epistasis inference case. Typically AUROC values rapidly increase and remain high over a broad time window. Recombination has little effect on the accuracy. MPL generally outperforms the single-locus (SL) estimator[40], and positive selection is inferred more accurately than negative selection.

bioRxiv preprint doi: https://doi.org/10.64898/2026.07.16.739011; this version posted July 20, 2026. The copyright holder for this preprint (which was not certified by peer review) is the author/funder, who has granted bioRxiv a license to display the preprint in perpetuity. It is made available under a CC-BY 4.0 International license.

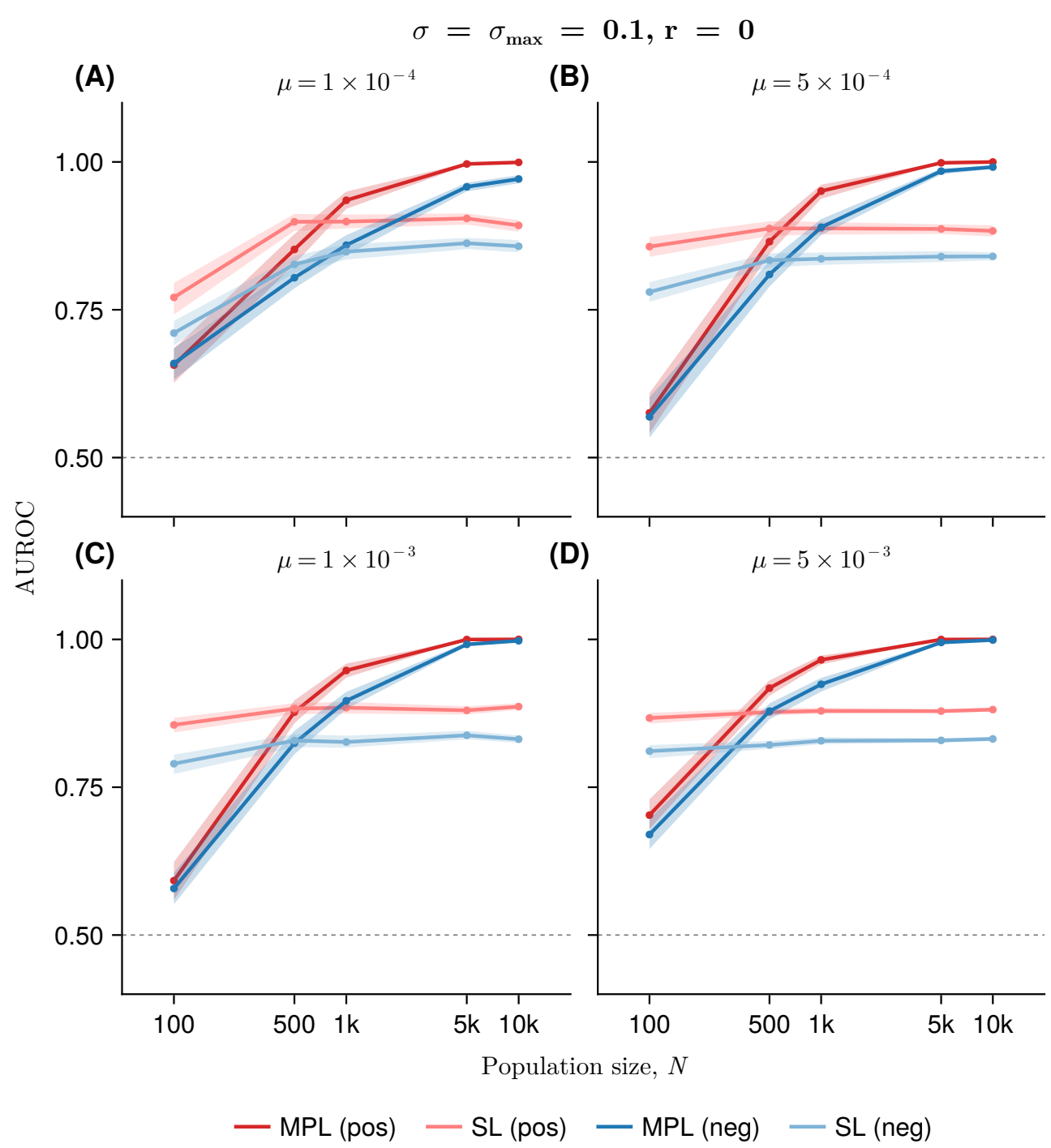

Supplementary Fig. S20. Effect of population size and mutation rate on selection-inference accuracy. AUROC values are computed at $t = 1000$. The SL estimator shows relatively weak dependence on population size N and tends to plateau at intermediate AUROC values. In contrast, MPL improves with increasing N, reaching 1.0 vlaue in large populations. The same qualitative pattern is observed across mutation rates).

bioRxiv preprint doi: https://doi.org/10.64898/2026.07.16.739011; this version posted July 20, 2026. The copyright holder for this preprint (which was not certified by peer review) is the author/funder, who has granted bioRxiv a license to display the preprint in perpetuity. It is made available under a CC-BY 4.0 International license.

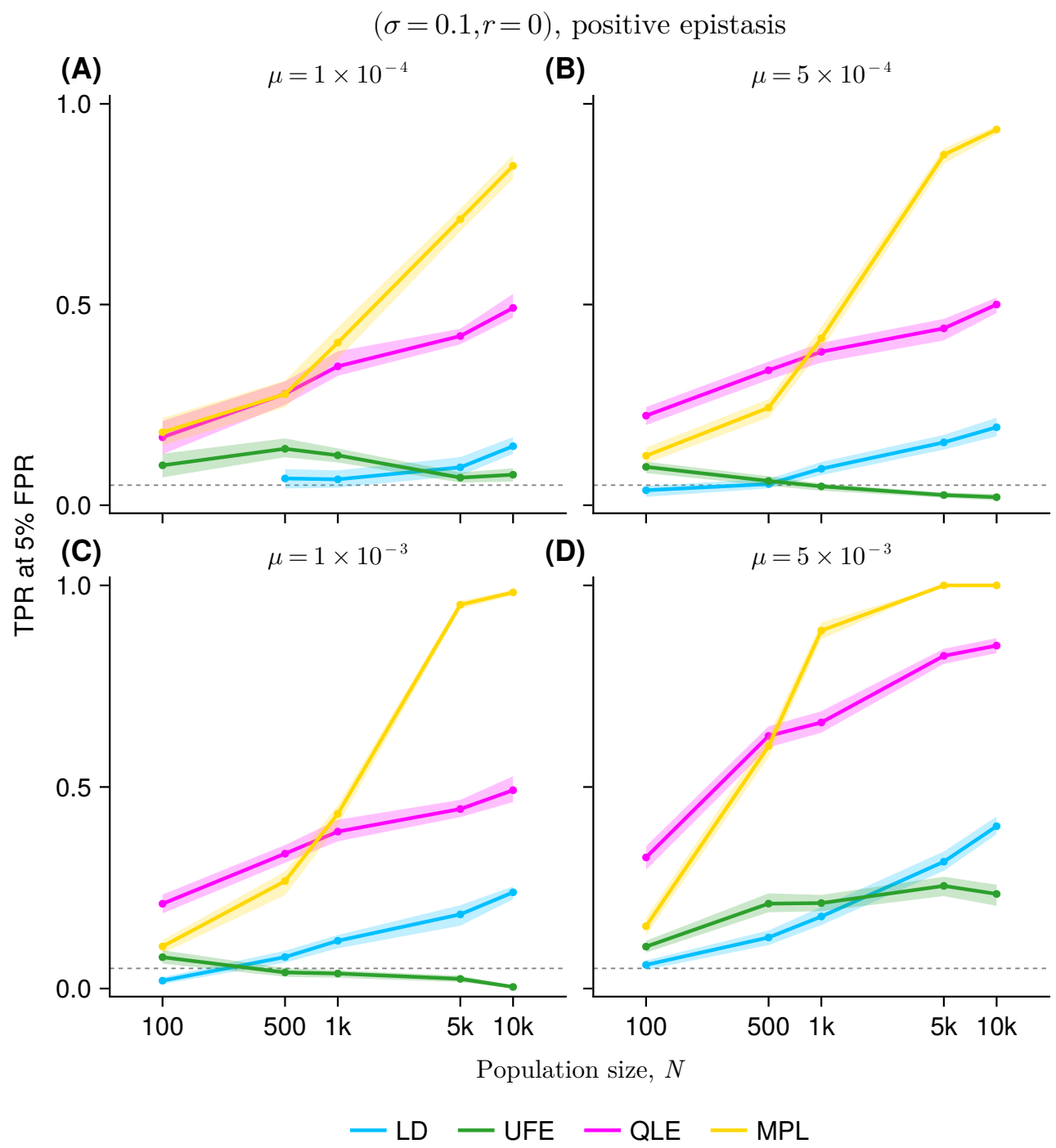

Supplementary Fig. S21. True-positive rate at a fixed false-positive rate as a measure of statistical power for epistasis detection. The true-positive rate at 5% false-positive rate (TPR@5% FPR) is shown as a function of population size for four mutation-rate conditions. Selection strength and recombination rate are fixed at $\sigma = 0.1$ and $r = 0$, respectively. Each curve shows the mean TPR@5% FPR across 30 replicate simulations, and shaded ribbons indicate the standard deviation. Consistent with the AUROC-based analysis in **Fig. 7**, inference accuracy generally increases with N and μ, particularly for QLE- and MPL-based approaches.

bioRxiv preprint doi: https://doi.org/10.64898/2026.07.16.739011; this version posted July 20, 2026. The copyright holder for this preprint (which was not certified by peer review) is the author/funder, who has granted bioRxiv a license to display the preprint in perpetuity. It is made available under a CC-BY 4.0 International license.

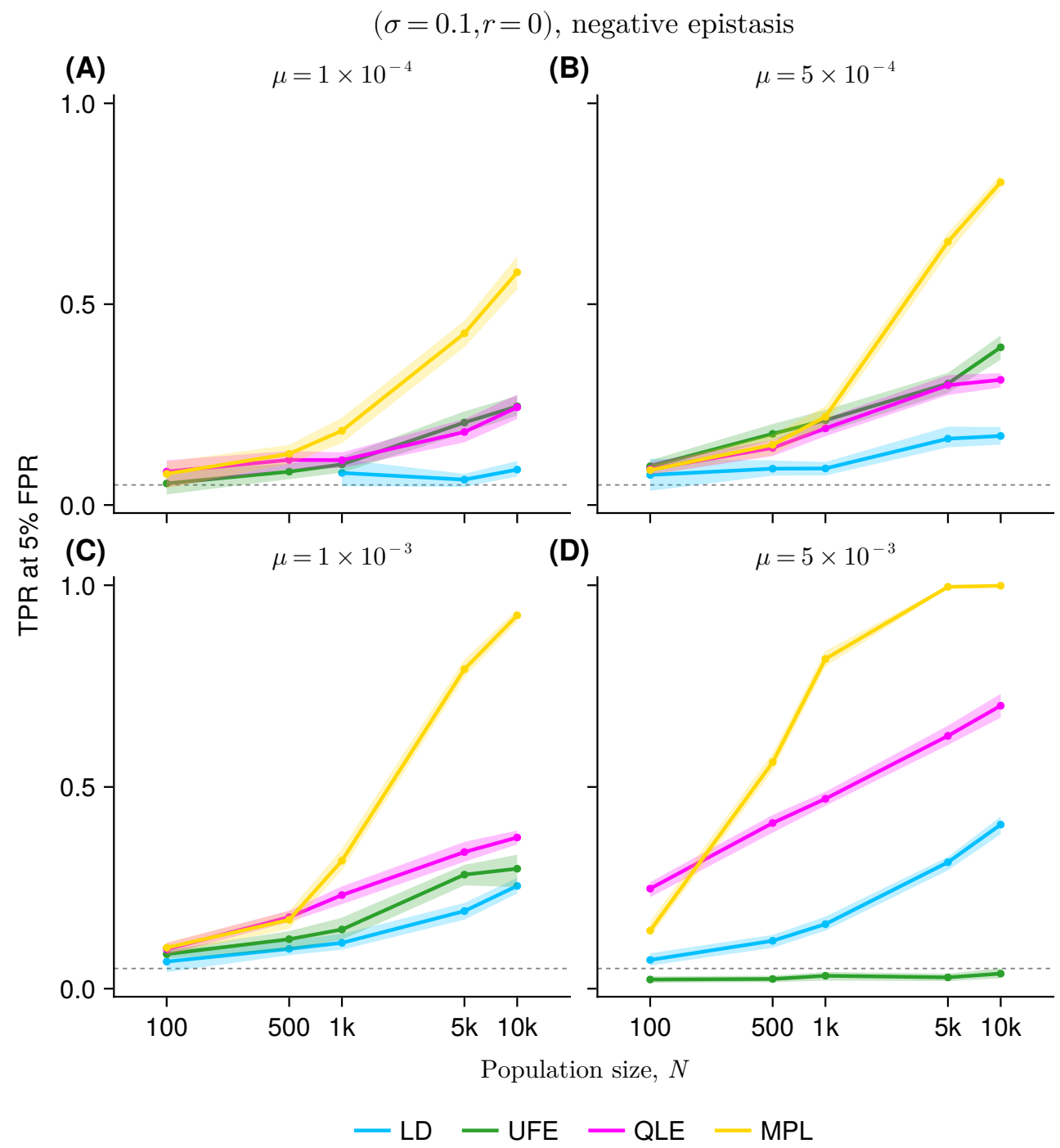

Supplementary Fig. S22. TPR@5% FPR is shown for detecting negative epistatic interactions as a function of population size N across multiple mutation rates μ. Although the absolute TPR@5% FPR values tend to be lower than those for positive epistasis, they are largely consistent with the positive epistasis case (**Supplementary Figure S21**).

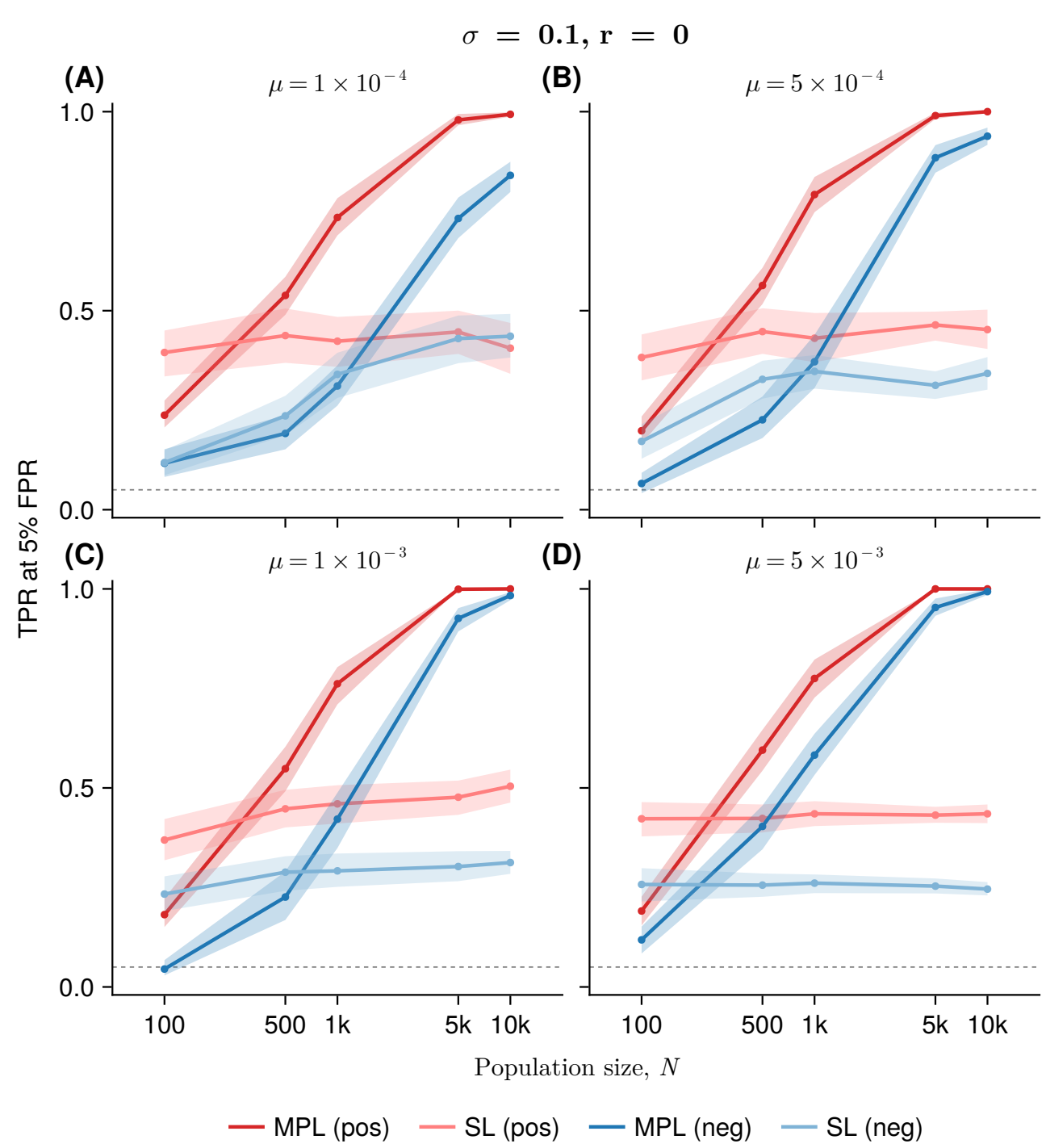

Supplementary Fig. S23. TPR@5% FPR for detecting positive and negative selection coefficients. The selection strength and recombination rate are fixed at $\sigma = 0.1$ and $r = 0$. Overall patterns are consistent with the AUROC-based analysis, but the difference between positive and negative cases is more pronounced under the TPR@5% FPR metric than under AUROC (**Supplementary Figure S20**).

bioRxiv preprint doi: https://doi.org/10.64898/2026.07.16.739011; this version posted July 20, 2026. The copyright holder for this preprint (which was not certified by peer review) is the author/funder, who has granted bioRxiv a license to display the preprint in perpetuity. It is made available under a CC-BY 4.0 International license.

	$\sigma = 0.01$			$\sigma = 0.04$			$\sigma = 0.10$		
t	pos/neu	neg/neu	pos/neg	pos/neu	neg/neu	pos/neg	pos/neu	neg/neu	pos/neg
50	0.051	0.053	0.082	0.120	0.118	0.230	**0.221**	0.137	**0.353**
200	**0.125**	**0.106**	**0.225**	**0.200**	**0.144**	**0.328**	0.145	**0.167**	0.297
1000	0.116	0.102	0.183	0.157	0.106	0.239	0.145	0.125	0.251

Supplementary Table 1. Kolmogorov-Smirnov (KS) test D statistics for LD distributions, $N = 10^3$, $r = 0$ (Fig. 3). The times that achieve the maximum D statistics among three time points are shown in bold. The subcolumns labeled pos/neg, neg/neu, and pos/neg represent the comparisons of the density of LD values for positive versus neutral epistasis, negative versus neutral, and positive versus negative, respectively. In the case of smaller selective pressure ($\sigma = 0.01$), the peak time occurs later. In contrast, for higher values of $\sigma = 0.1$, the peak time often appears earlier. For an intermediate selection strength of $\sigma = 0.04$, the peak time is slightly later. Overall, increases in σ lead to earlier maximum D values and greater separation of the distributions.

	$\sigma = 0.01$			$\sigma = 0.04$			$\sigma = 0.10$		
t	pos/neu	neg/neu	pos/neg	pos/neu	neg/neu	pos/neg	pos/neu	neg/neu	pos/neg
50	0.091	0.090	0.175	**0.315**	0.222	**0.502**	**0.406**	**0.318**	**0.623**
200	**0.376**	0.310	**0.635**	0.270	**0.232**	0.480	0.353	0.284	0.559
1000	0.327	**0.336**	0.567	0.207	0.193	0.397	0.152	0.229	0.373

Supplementary Table 2. KS test D statistics for LD distributions, $N = 10^4$, $r = 0$. Overall, the trends observed are similar to those seen in the small population size scenario. However, the time at which the highest D values are achieved occurs earlier among the three time points. The D values for the cases with a population size of $N = 10{,}000$ are generally larger than those for the smaller population sizes (**Supplementary Table 1**), suggesting an enhanced separation of distributions in larger populations.

	$\sigma = 0.01$			$\sigma = 0.04$			$\sigma = 0.10$		
t	pos/neu	neg/neu	pos/neg	pos/neu	neg/neu	pos/neg	pos/neu	neg/neu	pos/neg
50	0.028	0.037	0.055	0.076	0.053	0.111	0.176	0.128	0.266
200	0.056	**0.061**	**0.115**	0.058	0.089	0.140	0.147	0.098	0.211
1000	**0.073**	0.056	0.085	**0.114**	**0.170**	**0.283**	**0.210**	**0.149**	**0.356**

Supplementary Table 3. KS test D statistics for LD distributions, $N = 10^3$, $r = 0.01$. In contrast to cases where recombination is absent (**Supplementary Tables 1-2**), the observed pattern in asexual evolution (increasing sigma leads to an earlier separation of the LD distributions of positive/neutral, negative/neutral, and positive/negative) becomes ambiguous; the time at which the maximum D values are reached occurs later in evolution overall. Additionally, D values tend to be lower than the asexual case (**Supplementary Table 1**) in the presence of recombination, particularly under weak selection.

bioRxiv preprint doi: https://doi.org/10.64898/2026.07.16.739011; this version posted July 20, 2026. The copyright holder for this preprint (which was not certified by peer review) is the author/funder, who has granted bioRxiv a license to display the preprint in perpetuity. It is made available under a CC-BY 4.0 International license.

Supplementary References

39. Neher, R. A., Vucelja, M., Mezard, M. & Shraiman, B. I. Emergence of clones in sexual populations. *Journal of Statistical Mechanics: Theory and Experiment* **2013**, P01008 (2013).

40. Sohail, M. S., Louie, R. H., McKay, M. R. & Barton, J. P. Mpl resolves genetic linkage in fitness inference from complex evolutionary histories. *Nature biotechnology* **39**, 472–479 (2021).
